# Prevalence and Factors Associated With Depression Among Antenatal and Postnatal Women Attending Government Health Clinics in Selangor, Malaysia: Protocol for a Cross-Sectional Study

**DOI:** 10.2196/63663

**Published:** 2025-12-02

**Authors:** Nor Asiah Muhamad, Nur Hasnah Ma'amor, 'Izzah 'Athirah Rosli, Nik Athirah Farhana Nik Azhan, Nurul Hidayah Jamalluddin, Fatin Norhasny Leman, Tengku Puteri Nadiah Tengku Baharudin Shah, Nurul Syazwani Misnan, Noor Harzana Harrun, Norliza Chemi, Norni Abdullah, Nurashikin Ibrahim, Norli Abd Jabbar

**Affiliations:** 1 Sector for Evidence-based Healthcare National Institutes of Health Ministry of Health Selangor Malaysia; 2 Family Medicine Pandamaran Health Clinic Ministry of Health Klang Malaysia; 3 Department of Psychiatry and Mental Health Kajang Hospital Ministry of Health Kajang Malaysia; 4 Department of Psychiatry and Mental Health Tengku Ampuan Rahimah Hospital Ministry of Health Klang Malaysia; 5 National Centre of Exellence for Mental Health Ministry of Health Cyberjaya Malaysia

**Keywords:** prevalence, risk factors, antenatal, prenatal, depression, Selangor

## Abstract

**Background:**

Maternal mental health concerns either during pregnancy or the postpartum period are a public health challenge. Depression, anxiety, and stress can lead to poorer outcomes in the antenatal and postpartum periods. There is evidence that the effect of stress, anxiety, and depression during pregnancy negatively affects fetal neurodevelopment and children’s developmental outcomes. Early diagnosis can improve treatment outcomes and prevent negative impacts on the mother and baby. Identifying risk factors, such as age, socioeconomic status, mental health history, and family dysfunction, and clinical manifestations are important for public health programs.

**Objective:**

This study aims to determine the prevalence of depression and associated risk factors among antenatal and postnatal women attending government health clinics in Selangor.

**Methods:**

A multicenter cross-sectional study will be conducted among antenatal and postnatal women attending government health clinics (*Klinik Kesihatan*) in Selangor, Malaysia, from August 1, 2024, to December 31, 2026. We will perform a simple random sampling in all 9 districts in Selangor to select 1 government health clinic in each district. The inclusion criteria for this study are women aged 18 years and older who are either pregnant or delivered a newborn or stillborn child within the preceding 6 weeks. We will use a published screening tool (Edinburgh Postnatal Depression Scale) to determine the level of depression among antenatal and postnatal women. We will also collect data on sociodemographic characteristics, obstetric factors, and psychosocial support.

**Results:**

We will obtain ethics approval from the relevant ethics boards prior to data collection. Data will be analyzed using SPSS version 26.0 (IBM Corporation) and we will conduct a descriptive analysis to determine the prevalence of depression. We will calculate the level of depression among antenatal and postnatal women and score it based on a previous study conducted in Malaysia, with a score of ≥12 indicating the presence of depression. The association between depression and risk factors will be determined by multiple logistic regression analysis. *P* values less than .05 will be considered statistically significant.

**Conclusions:**

Depression is one of the mental health complications that may arise following childbirth. Therefore, the findings of this study on the prevalence and associated risk factors of depression among antenatal and postnatal women in Selangor may help women address this challenge and improve maternal mental health during pregnancy and after birth.

**International Registered Report Identifier (IRRID):**

PRR1-10.2196/63663

## Introduction

Early childhood is a critical period for brain development, and maternal depression can have a detrimental effect during this time. A few studies show that women are particularly susceptible to depression during the antenatal and postnatal periods. Depression can be due to hormonal changes and various psychosocial factors [1]. As a result, depression is more frequently reported in pregnant women than in those who are not pregnant [2]. To help mothers regain their prepregnancy health and well-being, they should receive adequate social support and care after childbirth. The postpartum period is a very important period for women, marked by significant transitions and instability, which require both adjustment and family support. However, some studies have shown that many women experience antenatal and postnatal depression during this period [3,4].

A study has shown that several pregnancies are unplanned or accidental, which can lead to negative attitudes toward pregnancy and increase the risk of prenatal depression in both Western and Asian cultures [5]. This attitude often manifests through symptoms such as exhaustion, anxiety, sleep disturbances, mood swings, irritability, emotional distress, sadness, and decreased self-esteem [4,6]. There is also substantial evidence indicating that maternal depression adversely affects a child’s physical health, cognitive development, and emotional well-being [7]. Additionally, women suffering from depression may experience poor prenatal care, which can lead to higher likelihood of obstetric complications, premature birth, and postpartum depression [8]. Even though a strong social support system occurs in many Asian cultures, both antenatal and postnatal depression remain common, with similar findings reported in Western countries [9].

Approximately 9% to 24% of pregnant women experience antenatal depression, while another 10% to 60% suffer from postnatal depression [5,10]. In Asia, the prevalence of antenatal depression stands at 20%, while postnatal depression ranges from 3.5% to 63.3% [5]. In Malaysia, a study found that 13.8% of women experienced antenatal depression and 14.3% reported postnatal depression [5]. These figures underline the need for greater attention to maternal mental health, as the rates of antenatal and postnatal depression are rising worldwide [3].

One of the primary factors contributing to both antenatal and postnatal depression is a lack of family support. Additionally, other factors, such as having more than 2 children, limited social support, conflicts with a partner or family, pre-existing mental health conditions, and experiencing intimate partner violence, may contribute to depression among pregnant mothers [11,12]. These issues can negatively affect personal well-being, marital satisfaction, social relationships, childcare, and child development, and may also prevent mothers from returning to work, which can impact the family’s financial stability [3,4,6]. Many working women are expected to return to their jobs within 3 to 6 months after childbirth, and some experience depressive symptoms during this time [13]. Additionally, parental depression has been shown to negatively affect an infant’s cognitive development, emotional growth, and behavioral outcomes [9].

Untreated depression can negatively affect both the mother and her unborn child, potentially affecting her ability to perform everyday tasks and damaging her relationships with family, colleagues or coworkers, and society. Prolong depression can also impact the child’s physical, social, psychological, and cognitive development [8]. During the postnatal period, the physical and emotional demands on the mother increase, and the disabling effects of postnatal depression may hinder her ability to care for and bond with her newborn and fulfil other maternal responsibilities [14]. In uncommon cases, the mother might become disengaged or even display negative behavior toward her child [7]. Depression during pregnancy and postpartum is a significant public health concern that requires continuous support from family, the community, health care providers, and government agencies. It has gained attention due to its detrimental impact on individuals, families, and child development and the global rise of the condition. Therefore, this study aims to identify the prevalence of depression and the associated risk factors among antenatal and postnatal women attending government health clinics in Selangor, Malaysia.

## Methods

### Study Design

This is a multicenter cross-sectional study to determine the prevalence of and risk factors associated with depression among antenatal and postnatal mothers attending government health clinics in Selangor, Malaysia.

### Study Population and Setting

This study will be conducted among antenatal and postnatal women attending government primary health clinics (*Klinik Kesihatan*) in Selangor, Malaysia from August 1, 2024, to December 31, 2026. The inclusion criteria for this study are women aged 18 years and older who are either pregnant or delivered a newborn or stillborn child within the preceding 6 weeks. The exclusion criteria are women who are not pregnant or have not deliver any newborn or stillborn child within the preceding 6 weeks. Patients who are diagnosed with a psychiatric illness, such as bipolar disorder, schizophrenia, or depression, during the study period will be excluded from this study. Since this study will not involve any biological sample collection, such as blood or urine, we do not anticipate providing any compensation to participants in the event of harm or loss resulting from their participation in the study. Additionally, all patient data will be kept confidential in both hard and soft copies.

### Recruitment and Data Collection

Selangor consists of 9 districts, of which 4 are in urban areas and 5 are in rural areas. In this study, we will identify all the major primary health clinics in each district. We will use a stratified random sampling method to identify 1 clinic in each district; hence, a total of 9 primary health clinics will be included. We will invite the family medicine specialist in the selected clinic to participate in this study.

In each selected primary health clinic, a liaison officer will be appointed to assist in the flow of recruitment of the respondents. We will conduct a training session prior to data collection. We will determine the respondents from the registration counter, and the respondents will be selected using a simple random sampling technique. When a respondent agrees to participate in this study, a face-to-face interview will be conducted by a trained interviewer, which may take 15 to 20 minutes. Verbal consent will be obtained prior to the interview.

Patients with a high score on the Edinburgh Postnatal Depression Scale (EPDS) will appropriately be referred to the family medicine specialist for further assessment and treatment.

### Sample Size

We will calculate the sample size using the following formula for estimating a proportion by OpenEpi software version 3.02, where n is the required sample size, Z is the z-score (Z=1.96 for 95% confidence), P is the estimated prevalence (P=0.09 [15]), and e is the margin of error (e=0.8):

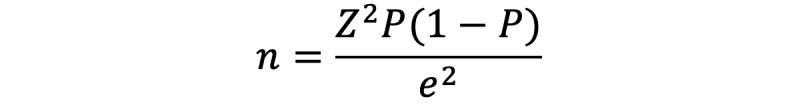



**Figure 1:**
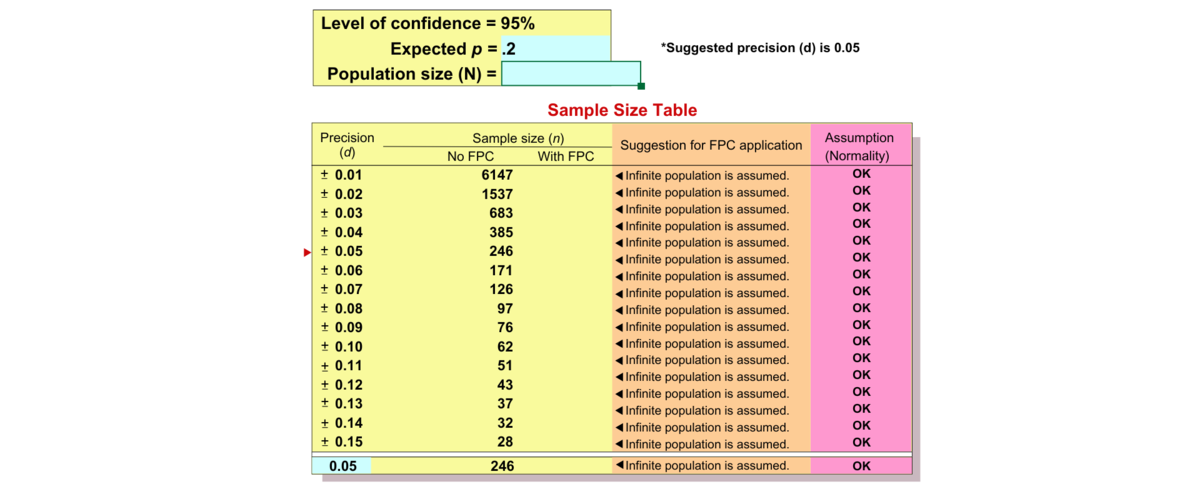
Sample size calculation for the prevalence study using random (not cluster) sampling. FPC: finite population correction.

A sample size calculation table is provided in Figure 1.

This gives a required sample size of 39 for each center. We will calculate the design effect using the formula DE = 1 / ICC, where ICC is the intraclass correlation and is estimated at 0.5.

The sample size will be adjusted using a design effect to account for the multicenter study design. We will estimate the nonresponse rate by anticipating that 20% of individuals will not agree to participate in this study. Therefore, we will increase the sample size by 10 to account for the anticipated nonresponse.

Our first objective is to determine the prevalence of depression among antenatal and postnatal women attending government health clinics in Malaysia. The prevalence of perinatal depression among pregnant women was 9% [15]. An expected prevalence of 0.09 and a precision of 0.05 are therefore taken into the calculation. With a 95% confidence level and 20% nonresponse rate, the study requires a minimum of 421 respondents.

**Figure 2:**
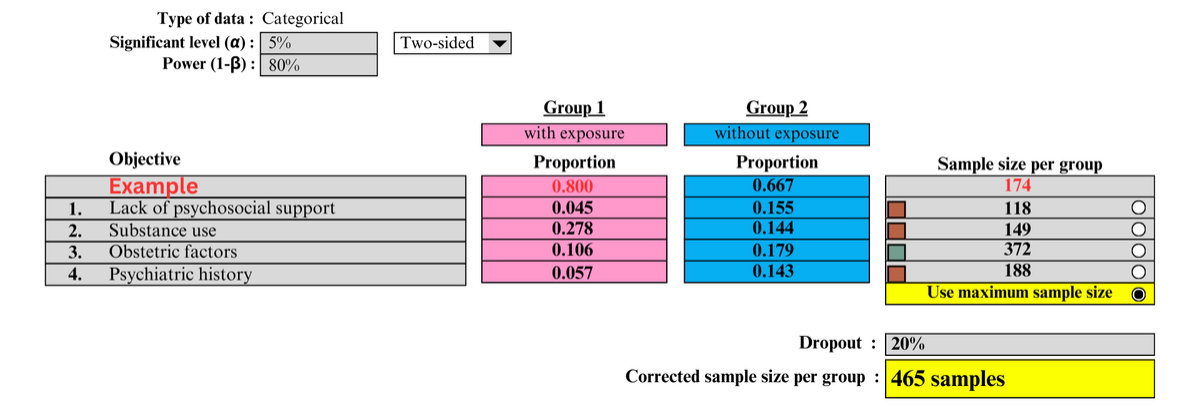
Sample size calculation for the associated risk factors among antenatal and postnatal women attending government health clinics in Malaysia.

Our second objective is to identify the risk factors for depression among antenatal and postnatal women attending government health clinics in Malaysia. Prior data indicate that the percentage of pregnant women with a lack of psychosocial support and who had a history of psychiatric illness were 8.6% and 28.3%, respectively [16]. The percentage of depressed pregnant women who had previous neonatal complications was 10.6% [17]. The proportion of antenatal women with depression who consumed alcohol was 27.8% [18]. Thus, a minimum sample of 372 respondents is needed. With an additional 20% to account for nonresponses, the sample size needed is 445. Therefore, we will use a minimum sample size of 445 respondents for this study (Figure 2).

### Research Tool

A guided interview by a trained interviewer will be conducted. A standardized web-based questionnaire consisting of 4 sections will be used, the details of which are provided below.

#### Section A: Sociodemographic Characteristics

This section consists of general information on the respondents’ age, education, economic status, marital status, employment, illness, history of substance use, and total current number of children.

#### Section B: Obstetric Factors

This section consists of information on obstetric conditions such as type of parity, history of abortion and preterm delivery, unplanned pregnancy, history of losing or hospitalization of baby, mode of delivery, place of delivery, pregnancy complications or illness, sex of the fetus, any uncomfortable symptoms faced during the confinement period, and stressful life events and social support during the antennal or postnatal period.

#### Section C: Psychosocial Support

This section will contain information on psychosocial factors. It includes partner relationship, emotional status (eg, worried, miserable, anxious, or depressed), any mental health concerns, support from other people to manage the baby, and experiences of sexual abuse, emotional, or physical abuse.

#### Section D: Edinburgh Postnatal Depression Scale

This scale consists of 10 screening questions designed to identify signs of depression and anxiety typical among pregnant and postpartum women. Respondents can select the number corresponding to how they have felt over the past week for each item. The scores for all 10 items are summed to calculate a total score. The EPDS has been previously validated in the Malay language, demonstrating high reliability with a Cronbach α of .86. Using a cutoff score of 11 or 12, the scale showed excellent diagnostic performance, with a sensitivity of 100%, specificity of 98.18%, positive predictive value of 90%, negative predictive value of 100%, and misclassification rate of only 1.56% [19].

In this study, we will use a cutoff score of 12 and above to indicate antenatal or postnatal depression based on the validation study conducted in a Malaysian population, which identified scores between 11 and 12 as indicative of depression [19]. The use of a lower cutoff score may be considered if the aim is to minimize false negatives and identify most patients who meet diagnostic criteria [20]. Prior to the main study, we will conduct a validity assessment, as the previous validation was carried out in a community-based setting, while our study will be conducted in a clinical setting. If respondents score 12 or above, their contact information will be collected and forwarded to the designated liaison officer for referral to the appropriate health care provider.

### Statistical Analysis

Data will be entered and organized into a Microsoft Excel spreadsheet by trained investigators. Data cleaning and verification will be conducted prior to analysis using SPSS software version 26.0 (IBM Corporation). We will perform a normality test to observe the distribution of the data. To assess the normality of data, we will plot a histogram to visualize the distribution, which is characterized by a bell-shaped curve for normal distributions, and perform a Kolmogorov-Smirnov test.

Descriptive analysis will be performed to summarize all variables, reporting means and SD. Result from the EPDS will be used to determine the prevalence depression. Chi-square analysis will be used for categorical variables to observe the association between depression and sociodemographic factors, obstetric factors, and psychosocial support. A multivariable logistics regression analysis will be conducted to identify the risk factors associated with depression among antenatal and postnatal women. Depression status (categorized as “depression” or “no depression”) will serve as the dependent variable, while associated risk factors, such as sociodemographic characteristics, obstetric factors, or psychosocial support, will be defined as independent variables. The logistic regression will use 3 methods, namely, enter, forward likelihood ratio, and backward likelihood ratio, to assess any differences in outcomes based on the method applied.

Multicollinearity among the predicted variables will be assessed using the variance inflation factor, which measures the strength and extent of correlation between independent variables in the regression model. All *P* values will be 2-sided, and a *P* value of less than .05 will be considered statistically significant.

### Ethical Approval

This study received ethics approvals from the Malaysian Research Ethics Committee (NMRR ID-24-01019-GZX) and Selangor State Medical Department.

We will keep the data on patients anonymized. Patient information will only be accessible to the principal investigator. Data will be kept in a private, password-protected database and will be linked only to a unique study identification number for the purposes of this research. Each patient will be assigned an ID number (eg, KK001) which will appear on all data collection forms. Upon completion of the study, the data will be transferred to an external soft copy, and the original files on the computer will be deleted. Both electronic and physical copies of the data will be securely stored in a locked cabinet that can only be accessed by the principal investigator. These records will be retained for a minimum of 3 years following the conclusion of the study, after which they will be permanently destroyed. The patients will not have access to their individual data, as the information will be integrated into a consolidated database.

## Results

Funded by the National Centre of Excellent for Mental Health, this will be a 2-year study (August 1, 2024, to December 31, 2026) from proposal development and data collection to analysis of the findings. Starting in June 2025, we will work with the selected primary health clinics in Malaysia, and we plan for data analysis to start in August 2025. The data will be analyzed using SPSS version 26.0 (IBM Corporation). We will share the findings with the public during a conference and publish the study in a reputable, peer-reviewed journal with a scope for maternal and depression research. This work will determine the prevalence of depression and its associated risk factors among antenatal and postnatal women referred to health care providers in Malaysia.

## Discussion

### Principal Findings

The incidence of depression among women before and after pregnancy has been steadily rising each year worldwide [11,12]. A study from the National Health and Morbidity Survey reported that 9 out of 10 mothers with depression were undiagnosed [21]. A study has shown that approximately 1 in 6 pregnant women experiences depression, underscoring the need for greater attention to specific risk factors [2]. When left untreated, maternal depression can have significant negative effects on the family members and the baby’s development during pregnancy [22] and may lead to preterm birth, low birth weight, and stillbirth [23]. It is also associated with negative health outcomes in children [22].

Antenatal depression significantly increases the risk of postpartum depression, with many postpartum cases being linked to depression during pregnancy [24]. Additionally, women with this condition are also at higher risk for substance abuse, premature rupture of membranes, severe headaches, and hemorrhage [25]. A previous report found that lacking social support; being single, separated, or divorced; having an unplanned pregnancy; being unemployed, experiencing violence, and smoking before or during pregnancy are also significantly associated with antenatal depression [25].

Therefore, understanding the factors contributing to depression are important for determining appropriate treatments or prevention strategies as well as developing awareness programs for pregnant women. Determining the prevalence of maternal depression may also help health care providers and policymakers implement targeted interventions, such as educational programs, psychological evaluation, and mental health assessments.

The findings of this study will be used to inform effective public health programs to reduce the incidence of maternal depression and serve as evidence to inform policy on improving antenatal and postnatal health care. The findings will be presented and published at academic conferences and in international peer-reviewed journals.

### Limitations

This is a cross-sectional study where we can only identify factors associated with depression among the population of antenatal and postnatal women. We are unable to make a conclusion with regard to cause and effect. However, due to the limited number of studies conducted within this specific population, our research may provide valuable information on the current state of depression among antenatal and postnatal women, especially in Malaysia.

### Conclusions

Depression is one of the mental health conditions that may arise following childbirth. The findings from this study will serve as a platform for the development of maternal mental health programs. These programs can be used for the early detection, prevention, and treatment of mental health conditions like depression, anxiety, and stress during antenatal and postnatal periods.

## Abbreviations

EPDS: Edinburgh Postnatal Depression Scale

## Data Availability

The data generated or analyzed during this study are available from the corresponding author upon reasonable request.

## References

[1] Sundström Poromaa I, Comasco E, Georgakis MK, Skalkidou A (2017). Sex differences in depression during pregnancy and the postpartum period. J Neurosci Res.

[2] Guo J, Zheng A, He J, Ai M, Gan Y, Zhang Q, Chen L, Liang S, Yu X, Kuang L (2021). The prevalence of and factors associated with antenatal depression among all pregnant women first attending antenatal care: a cross-sectional study in a comprehensive teaching hospital. BMC Pregnancy Childbirth.

[3] Glavin K, Leahy-Warren P (2013). Postnatal depression is a public health nursing issue: perspectives from norway and ireland. Nurs Res Pract.

[4] Teissedre F, Chabrol H (2004). A study of the Edinburgh Postnatal Depression Scale (EPDS) on 859 mothers: detection of mothers at risk for postpartum depression. Encephale.

[5] Mohamad Yusuff AS, Tang L, Binns CW, Lee AH (2016). Prevalence of antenatal depressive symptoms among women in Sabah, Malaysia. J Matern Fetal Neonatal Med.

[6] Babatunde T, Moreno-Leguizamon C (2012). Daily and cultural issues of postnatal depression in african women immigrants in South East london: tips for health professionals. Nurs Res Pract.

[7] Slomian J, Honvo G, Emonts P, Reginster J, Bruyère O (2019). Consequences of maternal postpartum depression: a systematic review of maternal and infant outcomes. Womens Health (Lond).

[8] Field T (2017). Prenatal depression risk factors, developmental effects and interventions: a review. J Pregnancy Child Health.

[9] Klainin P, Arthur DG (2009). Postpartum depression in Asian cultures: a literature review. Int J Nurs Stud.

[10] Hong SA, Buntup D (2023). Maternal depression during pregnancy and postpartum period among the Association of Southeast Asian Nations (ASEAN) countries: a scoping review. Int J Environ Res Public Health.

[11] Xayyabouapha A, Sychareun V, Quyen BTT, Thikeo M, Durham J (2022). Prevalence and risk factors associated with postpartum depressive symptoms among women in Vientiane Capital, Lao PDR. Front Public Health.

[12] Prabhu S, Guruvare S, George LS, Nayak BS, Mayya S (2022). Prevalence and associated risk factors of antenatal depression among pregnant women attending tertiary care hospitals in South India. Depress Res Treat.

[13] Majorie Ensayan Anak J, Cheah WL, Helmy H (2023). Corrigendum to "Postpartum health of working mothers: a prospective study". Malays Fam Physician.

[14] Arifin SRM, Cheyne H, Maxwell M (2018). Review of the prevalence of postnatal depression across cultures. AIMS Public Health.

[15] Pereira P, Lovisi G, Lima L, Legay L, de CSJ, Santos S, Thiengo D, Valencia E, Uehara T (2011). Depression during pregnancy: review of epidemiological and clinical aspects in developed and developing countries. Psychiatric Disorders - Trends and Developments.

[16] Nyamukoho E, Mangezi W, Marimbe B, Verhey R, Chibanda D (2019). Depression among HIV positive pregnant women in Zimbabwe: a primary health care based cross-sectional study. BMC Pregnancy Childbirth.

[17] Nielsen Forman D, Videbech P, Hedegaard M, Dalby Salvig J, Secher NJ (2000). Postpartum depression: identification of women at risk. BJOG.

[18] Chandrasekaran N, De Souza LR, Urquia ML, Young B, Mcleod A, Windrim R, Berger H (2018). Is anemia an independent risk factor for postpartum depression in women who have a cesarean section? A prospective observational study. BMC Pregnancy Childbirth.

[19] Mahmud WMRW, Awang A, Mohamed MN (2003). Revalidation of the Malay version of the Edinburgh Postnatal Depression Scale (EPDS) among Malay postpartum women attending the Bakar Bata Health Center in Alor Setar, Kedah, North West Of Peninsular Malaysia. Malays J Med Sci.

[20] Levis B, Negeri Z, Sun Y, Benedetti A, Thombs BD, DEPRESsion Screening Data (DEPRESSD) EPDS Group (2020). Accuracy of the Edinburgh Postnatal Depression Scale (EPDS) for screening to detect major depression among pregnant and postpartum women: systematic review and meta-analysis of individual participant data. BMJ.

[21] (2023). Maternal and child health. Ministry of Health Malaysia.

[22] Smith A, Twynstra J, Seabrook JA (2019). Antenatal depression and offspring health outcomes. Obstet Med.

[23] Jahan N, Went TR, Sultan W, Sapkota A, Khurshid H, Qureshi IA, Alfonso M (2021). Untreated depression during pregnancy and its effect on pregnancy outcomes: a systematic review. Cureus.

[24] Dlamini LP, Amelia VL, Shongwe MC, Chang P, Chung M (2023). Antenatal depression across trimesters as a risk for postpartum depression and estimation of the fraction of postpartum depression attributable to antenatal depression: a systematic review and meta-analysis of cohort studies. Gen Hosp Psychiatry.

[25] Yin X, Sun N, Jiang N, Xu X, Gan Y, Zhang J, Qiu L, Yang C, Shi X, Chang J, Gong Y (2021). Prevalence and associated factors of antenatal depression: systematic reviews and meta-analyses. Clin Psychol Rev.

